# Alopecias: Practical Tips for the Management of Biopsies and Main Diagnostic Clues for General Pathologists and Dermatopathologists

**DOI:** 10.3390/jcm12155004

**Published:** 2023-07-29

**Authors:** Fernando Pinedo-Moraleda, Belén Tristán-Martín, Giulia Greta Dradi

**Affiliations:** 1 Department of Pathology, Hospital Universitario Fundacion Alcorcon, 28922 Alcorcon, Spain; 2 Department of Pathology, Hospital Nuestra Señora de Sonsoles, 05004 Avila, Spain; btristan@saludcastillayleon.es; 3 Department of Dermatology, Hospital Universitario Fundacion Alcorcon, 28922 Alcorcon, Spain; giuliagreta.dradi@salud.madrid.org

**Keywords:** alopecia, cicatricial alopecia, follicular cycle, follicle histopathology, hair, hair loss, noncicatricial alopecia, nonscarring alopecia, scarring alopecia

## Abstract

Accurately diagnosing specific variants of alopecia remains challenging for pathologists, potentially delaying appropriate therapeutic decisions by dermatologists. Issues such as limited knowledge on optimal biopsy types and processing methods add complexity. Understanding the normal histology of hair follicles and their changes throughout the follicular cycle further complicates microscopic evaluation. This paper aims to summarize these characteristics and highlight essential diagnostic clues for pathologists to confidently suggest a diagnosis, therefore playing a key role in alopecia diagnosis. Ongoing education, collaboration with dermatologists, and staying up to date on advancements is crucial for the accurate diagnosis and effective management of different types of alopecia.

## 1. Hair Types

Hair loss poses a significant health concern for individuals affected by it. Not only does it impact their appearance but it also indicates underlying health issues, such as a heightened risk of coronary artery disease, hypertension [1], prostate cancer [2], and, more recently, a poorer prognosis for COVID patients [3,4]. Consequently, hair biopsy presents a challenge for pathologists, both general and dermatopathologists. The primary purpose of this article is to provide a comprehensive overview of the key aspects concerning hair follicle histology and the follicular cycle and offer practical guidance on managing hair biopsies. Additionally, the article aims to equip pathologists with the essential parameters for diagnosing the most common types of alopecia, hopefully enabling them to approach these biopsies with more confidence.

Hair can be classified into three types [5,6]: lanugo, vellus, and terminal hair. Lanugo hair is a fine and soft hair that covers the fetus during development and typically disappears after birth. Vellus hair is short, measuring less than 1 cm in length, and its hair bulb is found in the upper dermis. The diameter of vellus hair shafts is less than 0.03 mm, with an internal root sheath that is either equal to or larger than the shaft diameter, and it lacks a medulla. Terminal hair, on the other hand, is long, measuring greater than 1 cm in length, and its hair bulb is located in the subcutaneous tissue. Terminal hair shafts have a diameter greater than 0.06 mm and are thicker than the internal root sheath possessing both a cortex and a medulla. Intermediate hair, with a diameter ranging from 0.03 to 0.06 mm, is considered part of the terminal hair group.

From a functional perspective [5], terminal hair can be further categorized into androgen-dependent areas (scalp, beard, chest, axillae, and pubic region) and androgen-independent areas (eyebrows and eyelashes). Vellus hair, on the other hand, is not influenced by androgens and is independent of its effects.

## 2. Hair Follicle Histology (Figure 1) [5,7,8,9,10]

Hair follicles consist of two main components: the upper segment, also known as the permanent or fixed segment, and the low segment, referred to as the transitory or mobile segment. The superior segment can be further divided into two parts: the infundibulum and the isthmus. The infundibulum extends from the opening in the epidermis to the point where it passes through the sebaceous duct. The isthmus begins at the insertion point of the arrector pili muscle and widens into a region called the bulge, which houses the hair follicle’s stem cells. The low segment of the hair follicle is divided into two sections: the trunk (stem) and the bulb. The trunk (stem) extends from the insertion of the arrector pili muscle to the end of the cornified part of the bulb. Beyond this point, every cell in the bulb becomes completely keratinized, lacking a nucleus (known as Adamson’s fringe A). The bulb, located below this region, stretches from the end of the cornified part to the base of the hair follicle.

**Figure 1:**
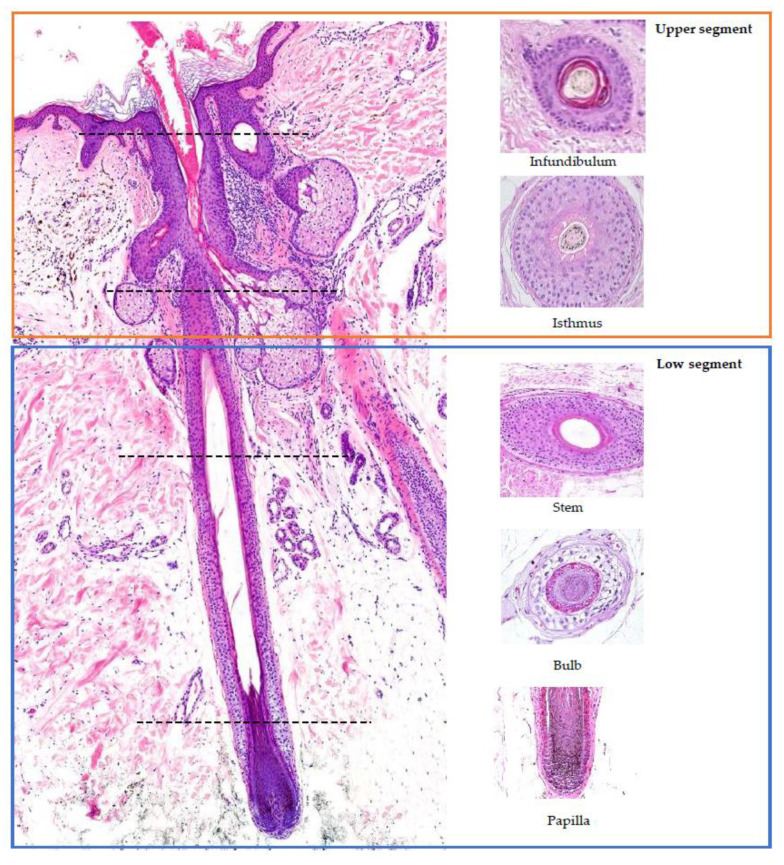
Histology of the hair follicle: vertical (**left**) and horizontal (**right**) sections. Red square: upper segment; blue square: low segment. The horizontal sections on the right correspond to the sectioning point marked by the line.

When examining a hair follicle in horizontal sections, which is crucial in the diagnosis of alopecia, several key structures can be observed. The bulb of the follicle contains the dermal papilla, which is responsible for providing blood supply and nutrients to the hair follicle through a central capillary. Additionally, the bulb consists of progenitor cells, dendritic melanocytes, the three layers of the internal hair sheath (Henle’s layer, Huxley’s layer, and the cuticle), the external root sheath (initially composed of one layer), and the vitreous membrane (basement membrane). At the higher levels of the follicle, a hair shaft consisting of three layers becomes visible: the cuticle, cortex, and medulla. The layers of the internal hair sheath merge into a single, fully keratinized layer, while the external root sheath increases in layers. In the upper layers of the trunk, the internal hair sheath undergoes desquamation (shedding), and the external root sheath becomes keratinized. At the level of the infundibulum, the hair follicle exhibits a degree of keratinization similar to that of the epidermis, characterized by a distinguishable granular layer and stratum corneum.

## 3. Hair Cycle (Figure 2) [5,8,9,11]

The only part of the hair follicle involved in the hair growth cycle is the lower segment. On the scalp, approximately 85 to 100% of hairs are typically found in the anagen phase, which lasts between 2 and 7 years. Hair follicles found in this phase correspond to the previously described histological features. When the hair follicle enters the catagen phase (which accounts for about 1% of hairs on the scalp, lasting around 2 to 3 weeks) it is characterized by the apoptosis of matrix cells, followed by thinning of the inferior segment. The vitreous membrane and the lower portion of the hair shaft thicken, while the hair becomes surrounded solely by the external root sheath, creating a “club hair” appearance. After the resting phase of the hair (telogen) is completed (which represents 0 to 15% of hairs on the scalp and lasts approximately 100 days), a new hair starts to replace the old one, initiating a new anagen phase.

**Figure 2:**
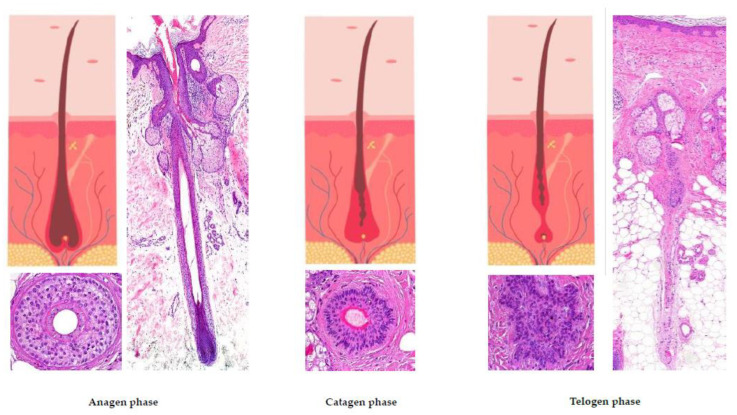
The hair growth cycle.

## 4. Adequate Hair Biopsy

Effective collaboration between dermatologists and pathologists is crucial in the assessment of skin biopsies, including those related to hair disorders. For a pathologist to accurately evaluate the biopsy, a detailed medical history and physical examination findings must be made available. This ensures a comprehensive diagnosis of the patient’s condition. Additionally, determining the appropriate biopsy site is essential for an accurate diagnosis. In cases of scarring alopecia, the biopsy should be taken from the active border of hair loss where inflammatory processes are present. If the biopsy is taken from the central part of the alopecia, it may only reveal cicatricial tissue replacing the hair shafts, making it difficult to determine the underlying cause. In non-scarring alopecia, it is important to identify and biopsy the active zone of the lesion. In cases of patchy alopecia, biopsies from the border are preferable, while in cases of diffuse hair loss, it is advisable to biopsy an area with a positive hair pull test [6,12]. For an optimal evaluation by a pathologist, the ideal biopsy should consist of two punch biopsies, each measuring 0.4 cm and taken obliquely, along the path of the hair follicle. These biopsies should extend into the subcutaneous tissue to enable a proper assessment of the hair bulb in terminal hairs. Smaller biopsies often do not provide sufficient material for an accurate diagnosis, while larger excision biopsies do not yield additional information and can be more challenging to process.

**Figure 3:**
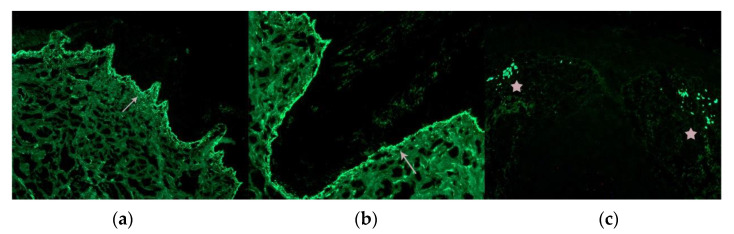
Direct immunofluorescence. Vertical sections: (**a**,**b**): Lupus erythematosus: (**a**) IgG granular deposits at the dermoepidermal junction (arrow) (IgG ×100) and (**b**) at the interface between the dermis and the follicular epithelium (arrow) (IgG ×200). (**c**): Lichen planopilaris: Necrotic keratinocytes (Civatte’s bodies) positive for IgM (star) (IgM ×100).

The availability of two 0.4 cm cylinders enables differential processing of the biopsy samples, allowing for both vertical (longitudinal) sections and horizontal (transverse) sections (Figure 3) [13,14,15]. Vertical sections are particularly useful for evaluating the epidermis and the dermoepidermal junction, which is important in conditions such as alopecia in the context of lupus erythematosus. This also facilitates the inclusion of a section for direct immunofluorescence study in cicatricial alopecia [16] (Figure 3). However, this technique may result in a lower number of observable hair follicles.

Horizontal sectioning, introduced by Headington in 1984 [17], allows for the evaluation of a greater number of hair follicles and enables the assessment of changes at different levels of the hair follicle [18]. At the infundibular level, follicular units can be observed. These units consist of three to five terminal hairs, one or two vellus hairs, the sebaceous gland, and the arrector pili muscle. Follicular units serve as the basis for the hair transplant technique known as follicular unit extraction (FUE). In the deep reticular dermis, terminal hairs are scarce, and only isolated vellus hair bulbs are visible. In the subcutaneous tissue, anagen-phase terminal hair bulbs can be observed, as well as the characteristic follicular stelae, typical of hairs in the telogen phase.

**Figure 4:**
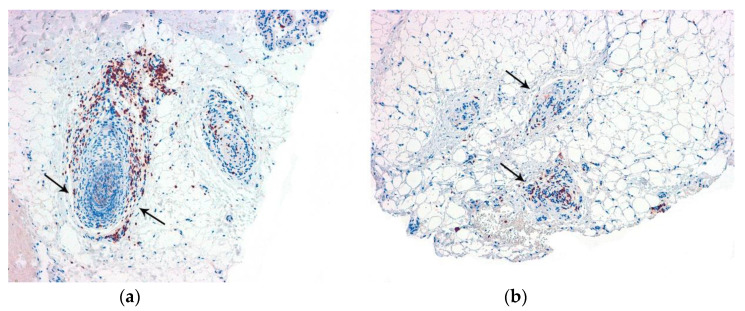
CD3. Alopecia areata: Positive T lymphocytes in the follicular streamers and peribulbar (arrows). (**a**) Vertical sections (CD3 ×100); (**b**) horizontal sections (CD3 ×100).

**Figure 5:**
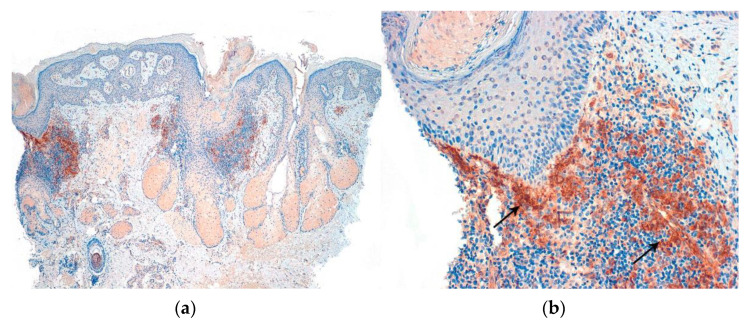
CD123. Lupus erythematosus: Positive aggregates of plasmocytoid dendritic cells. (**a**) Vertical sections (CD123 ×40); (**b**) Vertical sections: (CD123 ×200) (arrows).

Several histochemical techniques can be employed in the evaluation of alopecia specimens, and some of them are particularly noteworthy [19]. Periodic acid–Schiff (PAS) and Grocott stains are used to assess alopecia in the context of fungal infections. These stains help visualize the presence of fungal elements within the hair follicles and surrounding tissues. Giemsa stain is utilized to enhance the visualization of premature desquamation (shedding) of the internal root sheath. This can be helpful in identifying specific patterns or abnormalities related to the hair follicle’s structure. Stains used for evaluating elastic fibers [20] (orcein and van Gieson) are valuable in identifying patterns that are characteristic of scarring (cradle cap scar in lichen planopilaris, cylinder-like in central centrifugal cicatricial alopecia, etc.). For some authors [21], polarized light microscopy is a useful tool in differentiating scars in cicatricial alopecia (which exhibit polarization) from the fibrous trails observed in androgenetic alopecia (which do not exhibit polarization). Other histochemical techniques made popular in recent years which are worth mentioning include the use of CD3 [22,23] (Figure 4) to identify T lymphocytes in long-standing alopecia areata, the use of CD123 [24,25] (Figure 5) to detect aggregates of plasmacytoid dendritic cells in cases of lupus erythematosus, and the use of the antitreponemal antibody staining in cases of alopecia associated with syphilis infection.

**Figure 6:**
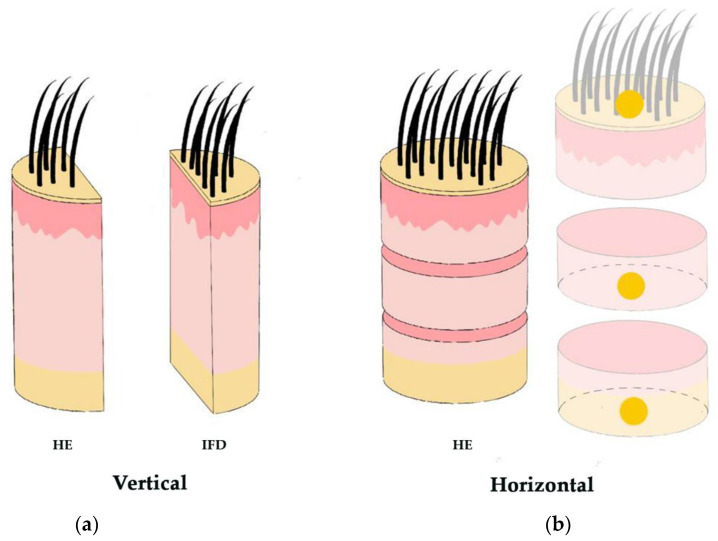
Biopsy processing technique. (**a**) Vertical sections: assessment of the epidermis and the dermoepidermal junction and inclusion for IFD study. (**b**) Horizontal sections: Greater number of hair follicles and evaluation of changes at different levels. The superior disc is marked at the apical pole and the two inferior discs are marked at the caudal pole.

Upon receiving the two biopsies, it is essential to ensure that both extend to the subcutaneous tissue. If one biopsy is superficial, the deeper one will be selected for horizontal sectioning. This selected specimen should be subjected to two cuts [18]: one just beneath the epidermis, approximately at the infundibulum, and the other at the junction between the deep reticular dermis and the subcutaneous tissue, resulting in three smaller specimens. The superior one should be marked with ink at the apical pole. As for the two inferior discs, they should be marked at the caudal pole and inserted into the cassette facing down (Figure 6). This approach allows for the progressive sectioning of the superior discs (from deep to superficial) and for the third specimen from the reticular dermis to the subcutaneous tissue. This method facilitates the evaluation of all structures at distinct levels, ensuring a comprehensive examination of the biopsy specimen. At least three consecutive histological sections are recommended for both vertical and horizontal cuts.

Lastly, when only one cylinder is available, a technique named HoVert [26] has been proposed, which involves vertical sectioning for the superior disc and horizontal sectioning for the two inferior discs when only one cylinder is available. The aim is to better examine the dermoepidermal junction. However, based on our experience, this technique does not offer any advantages and can potentially result in sample wastage if processing is not performed correctly. This is due to the fact that the 4 mm punch biopsy available in Europe is smaller than the ones available in the United States [27]. Overall, it is crucial that the processing of the biopsy material be handled by expert technicians [28].

## 5. Alopecia Classification

The classical distinction between nonscarring and scarring alopecia remains relevant in current practice. However, it is crucial to acknowledge that certain non-cicatricial alopecias can exhibit a biphasic nature and progress to scarring alopecia over time. This article aims to review the key findings of the most prevalent alopecias, enabling accurate diagnosis. While comprehensive monographs covering all types of alopecia are available, we strongly encourage our readers to explore these resources in order to gain a more in-depth understanding.

### 5.1. Nonscarring Alopecia [29]

Nonscarring alopecia encompasses various types, wherein the hair follicles can typically resume normal activity once the inflammatory process subsides [6]. The most frequent types include androgenetic alopecia, telogen effluvium, alopecia areata, trichotillomania, and traction alopecia.

A. **Androgenetic alopecia** [30,31,32]

This type occurs due to a combination of hormonal and genetical factors. In genetically predisposed individuals, an increase in the activity of the 5-alpha-reductase enzyme in the hair follicles causes them to miniaturize and shrink over time. This occurs especially on the temples, crown, and frontal regions, eventually resulting in bald patches or a receding hairline. Hair follicles in the occipital area are less affected, making it a suitable donor site for hair transplants.

**Figure 7:**
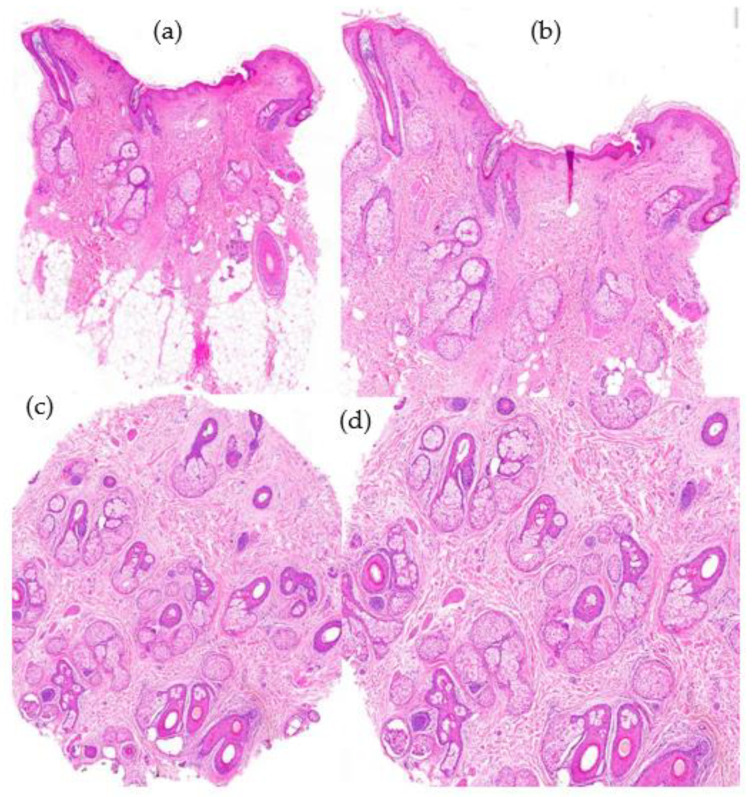
Androgenetic alopecia. (**a**) top left; (**b**) top right; (**c**) bottom left; (**d**) bottom right. (**a**,**b**) Vertical sections: miniaturization of terminal hair follicles and pseudohypertrophy of sebaceous glands (HE ×20; HE ×40). (**c**,**d**) Miniaturization of terminal hair and increase in telogen index with absence of perifollicular fibrosis (HE ×20; HE ×40).

1. Clinical Presentation:
  - Male pattern hair loss: characterized by bitemporal hairline recession, followed by the loss of hair in the frontotemporal and vertex regions.
  - Female pattern hair loss: typically manifests as diffuse hair loss, primarily affecting the central part of the scalp.

2. Histological Features (Figure 7):
  - Increase in the vellus index: miniaturization of terminal hair.
  - Increase in the telogen index.
  - Sebaceous gland pseudohyperplasia.
  - Perifollicular lymphocytic infiltrate (70%).
  - Absence of concentric fibrosis.
  - Polarized light: negative birrefringence of follicular streamers/stelae.

B. **Telogen effluvium** [33,34]

Telogen effluvium is a form of diffuse alopecia characterized by a sudden transition of a significant number of hair follicles from the anagen (growth) phase to the telogen (resting) phase. This condition can be triggered by various factors, including psychological stress, medication, infections, and other systemic diseases. Telogen effluvium is the most common type of hair loss associated with systemic conditions. It is advisable to perform the biopsy in the initial phases since the hair follicles restart the follicular cycle and biopsies may not reveal any abnormalities.

**Figure 8:**
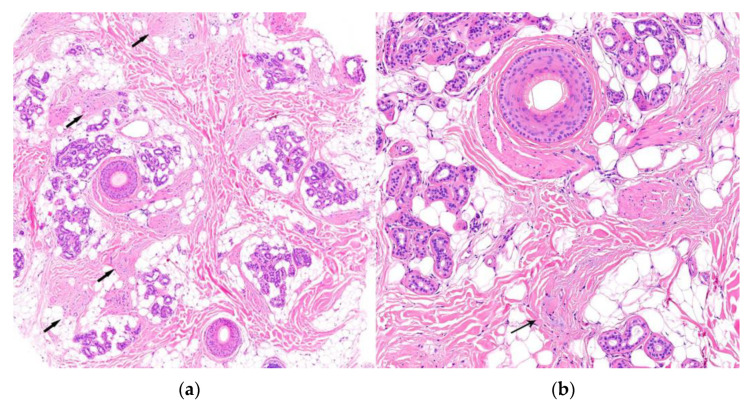
Telogen effluvium. (**a**,**b**) Horizontal sections. Increase in the telogen index with an increase in follicular streamers (arrow) and absence of perifollicular inflammation (HE ×20; HE ×100).

1. Clinical Presentation:
  - Diffuse alopecia.
  - Acute or chronic (if the diffuse hair loss has lasted for more than 6 months).
  - Can be associated with androgenetic alopecia, especially in males; for this reason it is advisable to perform a biopsy from the occipital area.

2. Histological Findings (Figure 8):
  - Increase in the telogen index (>25% in initial phases).
  - Absence of inflammatory infiltrate.
  - Normal terminal and vellus hairs with an increase in follicular streamers in horizontal sections.
  - Differential diagnosis between chronic telogen effluvium and female pattern hair loss. In the former, the telogen/anagen ratio is 8:1; in the latter, it does not exceed 4:1.

C. **Alopecia areata** [35,36,37]

Alopecia areata is an organ-specific autoimmune disease that affects approximately 1% of the general population, with a higher prevalence among children and young adults. This condition is genetically influenced and is characterized by an immune-mediated response primarily involving T lymphocytes (CD4+) targeting the keratinocytes of the hair follicles.

**Figure 9:**
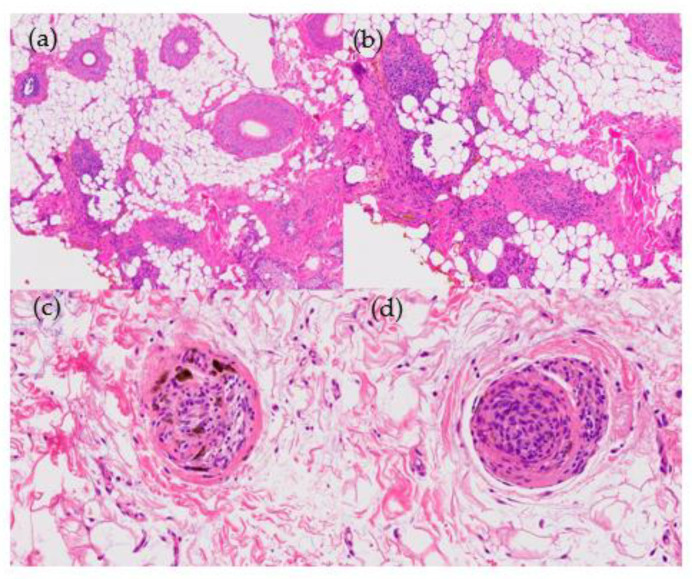
Alopecia areata. (**a**) top left; (**b**) top right; (**c**) bottom left; (**d**) bottom right. Horizontal sections. (**a**) Increase in vellus and telogen index (HE ×20) (**b**) Peribulbar lymphoid infiltrate during anagen phase (HE ×100). (**c**,**d**) Lymphocytes and melanin in follicular streamers (HE ×200).

1. Clinical Presentation:
  - Alopecia areata typically presents as patchy hair loss characterized by one or more circumscribed plaques on the scalp or other hair-bearing areas. The affected areas of the scalp usually exhibit underlying normal skin without any signs of inflammation or scarring.
  - One notable feature in alopecia areata is the presence of “exclamation mark” hairs. These are short, broken hairs that taper at the base and are commonly found at the borders of bald patches.
  - Can involve the whole scalp (total alopecia areata) or entire body (universal alopecia areata).

2. Histological Features (Figure 9):
  - Peribulbar inflammatory infiltrate: during the active phase of alopecia areata, a characteristic peribulbar inflammatory infiltrate is seen around the anagen (growth) hair follicles (“swarm of bees”).
  - Apoptosis of matrix cells within the hair follicle can be observed.
  - Presence of lymphocytes, eosinophils, and melanin in follicular streamers (inactive phase). Utility of CD3 staining.
  - Increase in vellus index.
  - Increase in telogen index.

D. **Trichotillomania** [38,39,40]

**Figure 10:**
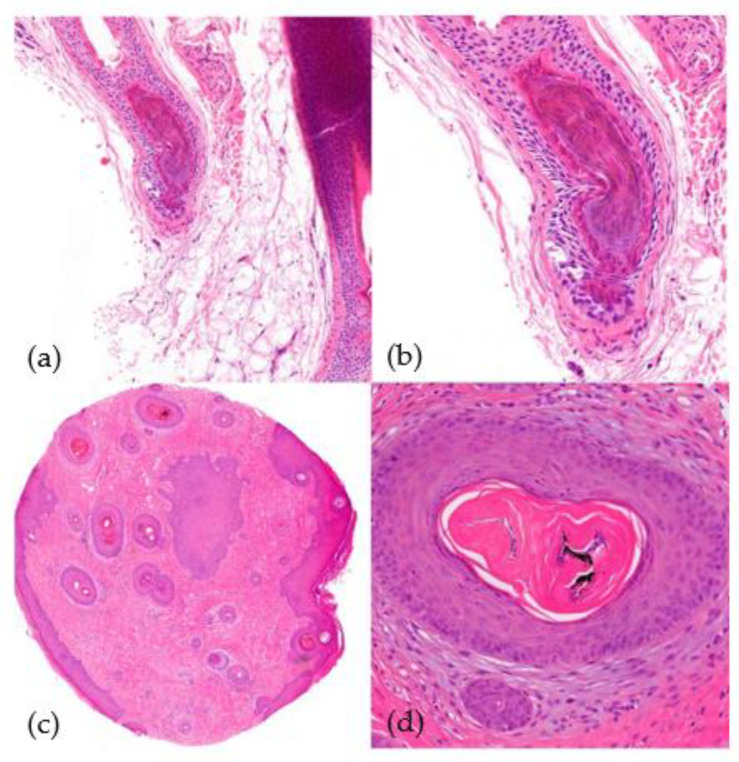
Trichotillomania. (**a**) top left; (**b**) top right; (**c**) bottom left; (**d**) bottom right. (**a**,**b**) Vertical sections. Distortion of the bulbar epithelium and trichomalacia (distortion of the hair shaft) (HE ×40; HE ×100). (**c**,**d**) Horizontal sections. Trichomalacia, pigmentary incontinence, and absence of inflammation (HE ×20; HE ×100).

1. Clinical Presentation:
  - Trichotillomania is characterized by a compulsive tendency, whether conscious or unconscious, to pull and twist one’s own hair.
  - Atypical patches of alopecia—these patches are typically irregular in shape and may appear as areas of partial or complete hair loss.
  - Presence of different hair lengths within the affected areas; the remaining hairs may appear frayed or have a jagged, uneven appearance.

2. Histological Findings (Figure 10):
  - Alternation of damaged and intact hair follicles.
  - Increased number of catagen hair follicles (>75%).
  - Bulbar epithelium distortion, hemorrhage, and pigmentary incontinence.
  - Trichomalacia (distortion of the hair shaft).

E. **Traction alopecia** [41,42]

1. Clinical Findings:
  - Form of alopecia caused by excessive inappropriate hair styling.
  - Hair loss occurs in areas that experience the most traction, especially the temples (frequently seen among black people).
  - Over time, it may transform into a cicatricial alopecia (known as follicular degeneration syndrome).

2. Histological Features:
  - - Similar to trichotillomania.

NOTE: Difficulty in distinguishing this from other types of alopecia such as alopecia areata and telogen effluvium [43].

### 5.2. Scarring Alopecias [29]

Scarring alopecias can be classified into two categories: primary and secondary. In primary scarring alopecias, the hair follicle itself is the target of the inflammatory process, leading to its destruction. In contrast, secondary scarring alopecias occur when the hair follicle is damaged within the context of other diseases or conditions; examples of this include infections such as tinea capitis (scalp ringworm) or neoplasms such as basal cell carcinomas.

The classification of scarring alopecias remains a topic of ongoing debate. In 2001, the North American Hair Research Society (NAHRS) proposed a classification system for primary cicatricial alopecias based on the predominant inflammatory cell observed in the biopsies of active lesions. According to this classification, primary cicatricial alopecias are categorized into four types: (1) lymphocytic, (2) neutrophilic, (3) mixed, and (4) non-specific [44]. This article aims to summarize the findings of the most prevalent alopecias, providing valuable insights for their diagnosis.

#### 5.2.1. Primary Scarring Alopecias [45,46]

A series of histological findings is common to all forms of primary scarring alopecia, such as the destruction of the hair follicle, replacement of fibrous tracts, loss of sebaceous glands, and dilation of eccrine ducts [47,48]; these are signs that a pathologist should look to find in these types of biopsies.

##### Associated to Lymphocytic Infiltrate [49]

Primary scarring alopecias associated with lymphocytic infiltrate include lupus erythematosus, lichen planopilaris (including frontal fibrosing alopecia, Graham-Little syndrome, and classic lichen planopilaris), pseudopelade of Brocq, central centrifugal cicatricial alopecia, alopecia mucinosa, and keratosis follicularis spinulosa decalvans. These conditions involve an immune-mediated inflammatory response predominantly involving lymphocytes, leading to scarring and irreversible hair loss. Folliculitis decalvans and dissecting cellulitis of the scalp (perifolliculitis capitis abscedens et suffodiens) are considered primary neutrophilic scarring alopecias. Mixed primary cicatricial alopecias encompass conditions such as acne keloidalis nuchae, necrotic acne (folliculitis), and erosive pustular dermatosis of the scalp.

A. **Lupus erythematosus (chronic cutaneous)** [15,50]

**Figure 11:**
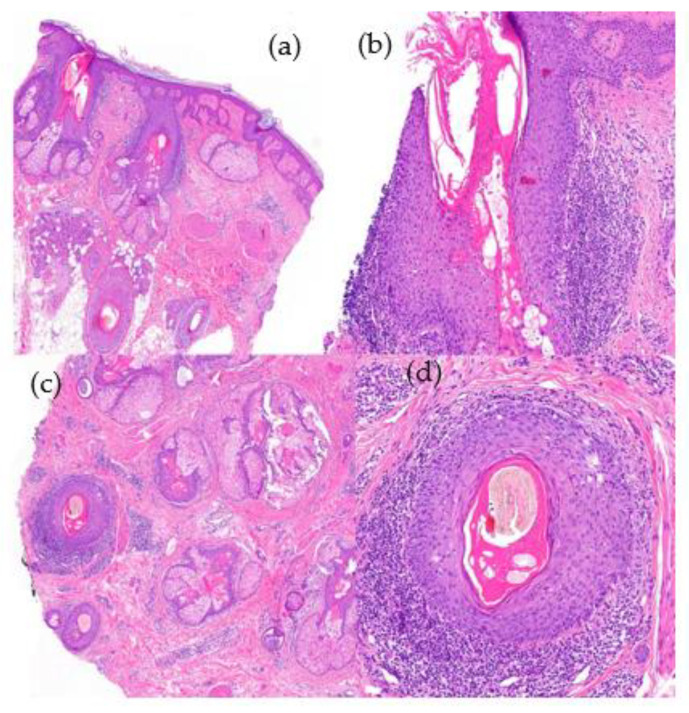
Alopecia in lupus erythematosus. (**a**) top left; (**b**) top right; (**c**) bottom left; (**d**) bottom right. Vertical sections. (**a**) Infundibular hyperkeratosis (HE ×20); (**b**) vacuolar interface dermatitis in the follicular epithelium (HE ×100). Horizontal sections. (**c**,**d**) Similar findings without perifollicular fibrosis and the absence of necrotic keratinocytes (HE ×20; HE ×100).

1. Clinical Presentation:
  - Affects approximately 50% of patients.
  - Middle-aged women; presenting as papules or erythematodesquamative plaques with associated pigmentary disorders, including hypo- and hyperpigmentation.
  - Follicular obliteration may occur.

2. Histological Features (Figure 11):
  - Hyperkeratosis predominantly involving the infundibulum of the hair follicle.
  - Vacuolar interface dermatitis; primarily affects the follicular epithelium and the dermoepidermal junction.
  - Presence of isolated Civatte’s bodies [51].
  - Superficial and deep perivascular and periadnexal lymphocytic infiltrate.
  - Pigmentary incontinence.
  - Increased dermal mucin.
  - Immunofluorescence (IFD) testing reveals a positive lupus band characterized by granular deposits of IgG, IgM, and/or C3 at the dermoepidermal junction and follicular epithelium.
  - Orcein staining reveals elastic fiber destruction throughout the entire dermis (advanced stages).

B. **Lichen planopilaris (LPP)**

This is a term used to describe the manifestation of lichen planus that specifically affects the hair follicles. As mentioned earlier, it encompasses classic LPP, frontal fibrosing alopecia, and Graham-Little syndrome, which share similar histological characteristics and are often challenging to differentiate. However, some authors argue that frontal fibrosing alopecia should be regarded as a distinct primary cicatricial alopecia due to its distinct clinical presentation, even though it shares histological features with classical LPP [52,53].

B.1. Classic LPP [15,50]

**Figure 12:**
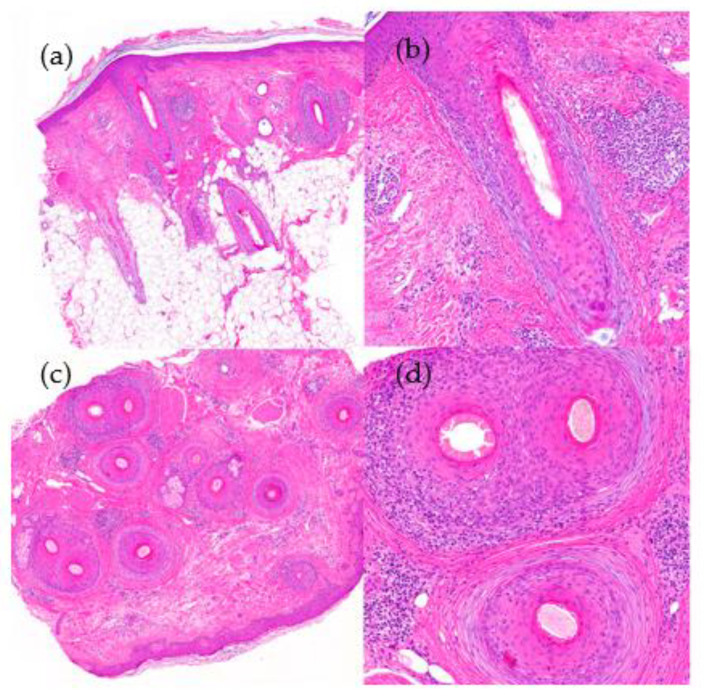
Lichen planopilaris. (**a**) top left; (**b**) top right; (**c**) bottom left; (**d**) bottom right. Vertical sections. (**a**,**b**) Interface dermatitis, lichenoid type, observed in the follicular epithelium with lymphocyte permeation (HE ×20; HE ×100). Horizontal sections. (**c**,**d**) Concentric perifollicular fibrosis, lymphocytic infiltrate, and necrotic keratinocytes (HE ×20; HE ×100).

1. Clinical Presentation:
  - Atrophic plaques with perifollicular hyperkeratosis and erythema; affects middle-aged women more frequently than men.

2. Histological Features (Figure 12):
  - Hypergranulosis and infundibular hyperkeratosis.
  - Lichenoid interface dermatitis observed in the follicular epithelium, specifically the infundibulum and isthmus, as well as at the dermoepidermal junction.
  - Lymphocytic infiltration of the follicular epithelium.
  - Presence of abundant Civatte’s bodies (necrotic keratinocytes) within the follicular epithelium (detectable through positive cytokeratin staining) [51].
  - Concentric perifollicular fibrosis (advanced stages) with retraction clefts.
  - Orcein staining reveals a cradle cap scar centered around the follicle.
  - Immunofluorescence (IFD) testing is positive for IgM deposits in the follicular epithelium.
  - IFD: the abundant Civatte bodies are frequently positive for IgM.

NOTE: A lymphocytic perifollicular infiltrate can also be found in other types of alopecia (for example, in androgenetic alopecia in up to 70% of cases).

B.2. Frontal fibrosing alopecia (FFA) [54,55,56,57,58]

1. Clinical Presentation:
  - Post-menopausal women but may be also seen in men and premenopausal women.
  - Regression of the frontemporal hairline and eyebrow loss.
  - Facial papules and in other body areas.

2. Histological Features:
  - Similar to classic LPP.
  - Facial [59,60,61] and extrafacial [62,63] papules; these signs are present around vellous hair.
  - “Follicular triad”—simultaneous involvement of terminal hair follicles, intermediate follicles, and vellus follicles at various stages of the hair follicle cycle (a key finding during the initial phases of the disease) [64].
  - Adipose infiltration of the arrector pili muscle and displacement of the eccrine glands [65].

B.3. Graham-Little syndrome [66,67]

1. Clinical Presentation:
  - Cicatricial alopecia of the scalp.
  - Presence of keratotic follicular papules on the trunk and extremities.
  - Reversible loss of pubic and/or axillary hair.

2. Histological Features:
  - Similar to that of LPP and FFA.

B.4. Fibrosing alopecia in a pattern distribution (FAPD) [68]

**Figure 13:**
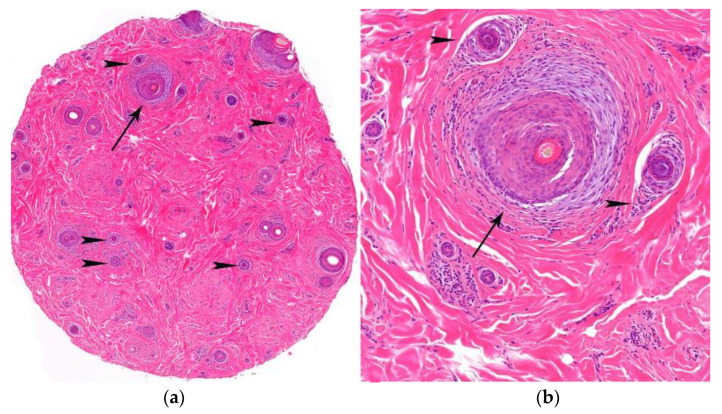
FAPD. (**a**,**b**) Horizontal sections. Increase in vellus index (hair miniaturization) (arrow heads) and lymphocytic infiltrate and concentric perifollicular fibrosis (arrows) (HE ×20; HE ×100).

1. Clinical Presentation:
  - Described by Zinkernagel an Trüeb in the year 2000 [69]; considered as an exaggerated inflammatory response to hair follicles affected by androgenetic alopecia.
  - It exhibits characteristics of both androgenetic alopecia and LPP.
  - Primarily affects the androgen-dependent areas of the scalp while sparing areas that are androgen-independent, such as the occipital region.
  - Perifollicular hyperkeratosis, loss of follicular ostium, and variation in hair shaft diameter are observed [70].

2. Histological Features (Figure 13):
  - Increase in vellus index (hair follicle miniaturization).
  - Lymphocytic perifollicular infiltrate (isthmus and infundibulum) with lamellar concentric perifollicular fibrosis [70].

C. **Pseudopelade of Brocq** [50]

Pseudopelade of Brocq, described by Brocq in 1885 [71], remains a topic of debate as to whether it represents a distinct entity or signifies the non-inflammatory end stage of other primary cicatricial alopecias [72,73]. For certain authors the diagnosis of this entity relies on excluding other primary cicatricial alopecias, such as LPP or cutaneous lupus [51].

**Figure 14:**
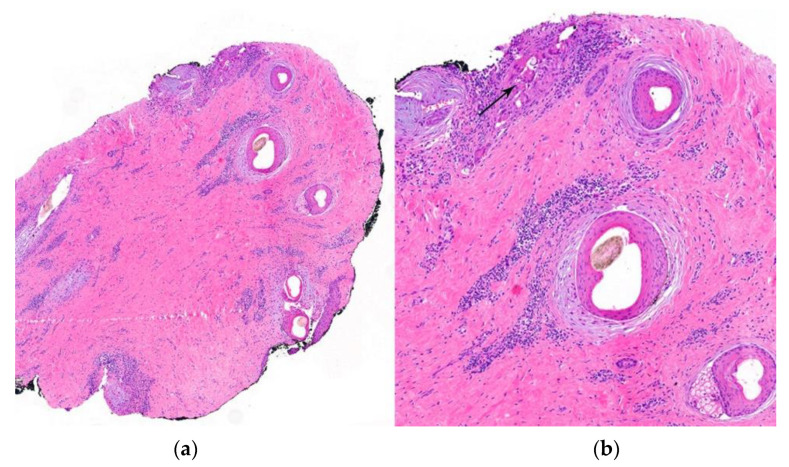
Pseudopelade of Brocq. (**a**,**b**) Horizontal sections. Loss of sebaceous glands with concentric fibroplasia and chronic inflammation around the hair follicles. (**b**) Foreign body reaction (arrow) around naked hair shafts (HE ×40).

1. Clinical Features:
  - Middle-aged women with small alopecic plaques with normal underlying skin. These plaques have irregular borders and are devoid of keratotic papules or perifollicular erythema.
  - Primarily affects the vertex and parietal areas of the scalp.

2. Histological Features (Figure 14):
  - No definitive histological criteria have been described. No interface dermatitis is seen.
  - Concentric fibroplasia centered around the hair follicles.
  - Loss of sebaceous glands with preservation of the arrector pili muscle.
  - Granuloma formation around the naked hair follicles.
  - Orcein staining reveals thickened elastic fibers in the adventitial and reticular dermis (differential diagnosis with LPP and lupus erythematosus) [20,74].
  - IFD is negative.

D. **Central centrifugal cicatricial alopecia (CCCA)** [50,75]

This is a descriptive term used to characterize scarring alopecias that originate in the vertex area and gradually progress in a centrifugal pattern, as described in the North American Hair Research Society’s (NAHRS) classification. This category encompasses various conditions, including follicular degeneration syndrome, pseudopelade in black people and central elliptic pseudopelade in white people [51]. Clinically, CCCA is distinct from pseudopelade of Brocq, but histologically, they share similarities.

**Figure 15:**
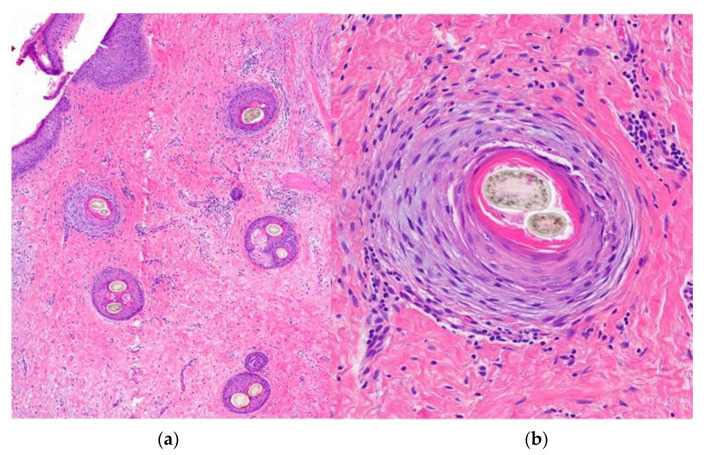
Central centrifugal cicatricial alopecia. Horizontal sections. (**a**) Concentric fibroplasia accompanied by sebaceous gland destruction (HE ×20). (**b**) Eccentric atrophy of the follicular wall with hair shaft duplication (HE ×100).

1. Clinical Presentation: see definition. More commonly seen among black people.
2. Histological Features (Figure 15):
  - For some authors, very similar to LPP [22,76].
  - Perifollicular lymphocytic infiltrate around the superior portion of the hair follicle.
  - Lamellar fibroplasia with sebaceous gland loss.
  - Atrophy of the follicular wall.
  - Duplication of hair shafts.
  - Premature desquamation of the internal root sheath (Giemsa staining).
  - Orcein staining: similar to pseudopelade of Brocq.

E. **Alopecia Mucinosa** [77,78,79]

Inflammatory process of the pilosebaceous follicle characterized by mucin deposition in the follicular epithelium. There is an idiopathic primary form, which is considered a premalignant condition or an indolent form of mycosis fungoides. This form is typically observed in children and young adults. Additionally, there are secondary forms associated with a type of cutaneous T-cell lymphoma (follicular mycosis fungoides), which are more common in older patients (approximately 30% of cases). In the primary form, the condition is usually self-limited but may result in permanent hair loss due to the complete destruction of the follicle.

**Figure 16:**
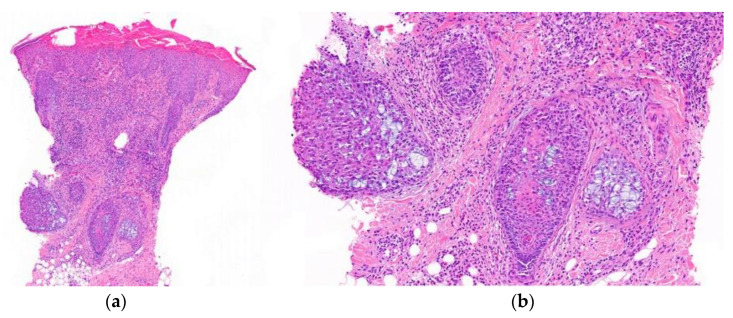
Alopecia mucinosa. Vertical sections. (**a**) Intense lymphocytic infiltrate observed both perifollicularly and intrafollicularly (HE ×20); (**b**) Follicular mucinosis characterized by mucin deposition and reticular degeneration affecting the entire follicle (HE ×100).

1. Clinical Presentation:
  - Predominant involvement of the head and neck in the form of grouped papules with a follicular distribution, erythematous patches, and/or fluctuating plaques, especially in the primary forms found in children and young adults [51].
  - Numerous lesions on the trunk and extremities can be seen in secondary forms and older patients.

2. Histological Features (Figure 16):
  - Follicular mucinosis: Mucin deposition initially affects the external root sheath and the infundibulum of the hair follicle [51]. In later stages, the entire hair follicle and sebaceous glands may be involved.
  - Lymphocytic infiltrate—there is a presence of lymphocytic infiltrate both peri and intrafollicularly.
  - Cytological atypia and monoclonal rearrangement in idiopathic and secondary forms.

F. **Keratosis follicularis spinulosa decalvans (KFSD)** [80]

X-linked genodermatosis characterized by widespread cicatricial alopecia, which affects various areas such as the scalp, eyebrows, eyelashes, and axillae. In addition to alopecia, individuals with KFSD may experience other associated symptoms, such as photophobia and keratoderma.

1. Clinical Presentation:
  - Patches of hair loss with follicular papules, hyperkeratosis, and pustules.

2. Histological Features [51]:
  - Abnormal keratinization with hypergranulosis and compact hyperkeratosis affecting the infundibulum, followed by spongiosis and neutrophilic infiltrate.
  - In later stages, chronic lymphocytic inflammation and fibrosis with a perifollicular distribution is observed.
  - In the final stages, destruction of the hair follicle with fibrosis and tricogranulomas can be observed.

##### Lichenoid Folliculitis

Lichenoid folliculitis is a term coined by Sperling in 2017 [45] used to encompass a group of cutaneous diseases characterized by fibrosis and chronic perifollicular inflammation. These conditions are characterized by the presence of keratotic papules, atrophy, scarring alopecia, and lichenoid changes upon histological examination. The diseases within the lichenoid folliculitis group can be divided into two main subgroups based on clinical characteristics. The first subgroup is keratosis pilaris atrophicans, which includes conditions such as keratosis pilaris atrophicans faciei/ulerythema ophryogenes, atrophoderma vermiculatum, and keratosis follicularis spinulosa decalvans. The second subgroup is the LPP subgroup, which includes classical LPP, FFA, Graham-Little syndrome, and FAPD. These subgroups share histological similarities and demonstrate significant clinical overlap.

##### Associated to Neutrophilic Inflammation

Group of alopecias characterized by acneiform dilation of the hair follicle and a neutrophilic inflammatory infiltrate. It primarily affects the follicular epithelium and the surrounding dermis, progressing to a mixed inflammatory infiltrate with fibrosis over time. Another similar condition in this category is the dissecting cellulitis of the scalp, but we will focus specifically on folliculitis decalvans in this article, as the histological findings are comparable in both conditions.

A. **Folliculitis decalvans** [81,82]

Suppurative destructive folliculitis.

**Figure 17:**
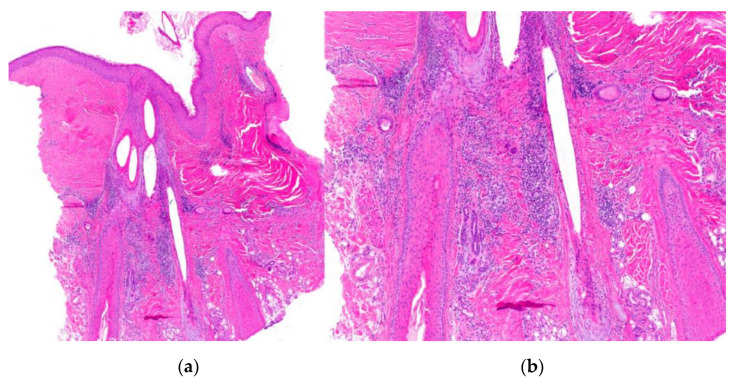
Folliculitis decalvans. (**a**,**b**) Vertical sections. Infundibular dilation accompanied by a polymorphous inflammatory infiltrate, both within and surrounding the hair follicle, characterized by polymorphonuclear leukocytes and multinucleated giant cells. (HE ×20; HE ×100).

1. Clinical Features:
  - Typically presents as alopecic patches with follicular pustules predominantly seen along the active borders.
  - More frequently around the crown, but it can also involve other regions, such as the beard, axilla, pubic area, arms, and legs.
  - Tufting, where multiple hairs emerge from a single hair follicle, is frequent.

2. Histological Features (Figure 17):
  - Infundibular dilation with peri- and intrafollicular neutrophilic infiltrate in early stages.
  - Polymorphous infiltrate in advanced stages (lymphocytes, plasma cells, histiocytes, and multinucleated giant cells).
  - Follicular loss and scarring.
  - Naked hair shafts.
  - Negative fungal stains (PAS, Grocott).
  - Involvement of the interfollicular dermis.

##### Mixed Primary Cicatricial Alopecias

Includes acne keloidalis nuchae, erosive pustular dermatosis, and acne necrotica.

#### 5.2.2. Secondary Scarring Alopecia [83]

Secondary scarring alopecia refers to a group of alopecias that occur as a consequence of other processes where the involvement of the hair follicle is incidental rather than the primary focus. There are various conditions that can fall into this category, including genodermatoses, physical or chemical trauma, inflammatory dermatoses, drug-induced alopecia, and neoplasms. In this discussion, we will focus specifically on tinea capitis.

A. **Tinea capitis** [84]

Fungal infection of the scalp that is predominantly seen in children. It is highly contagious and can spread rapidly, leading to epidemics in certain settings. The infection is commonly caused by two types of fungi: Trichophyton tonsurans (endothrix) and Microsporum canis (ectothrix).

**Figure 18:**
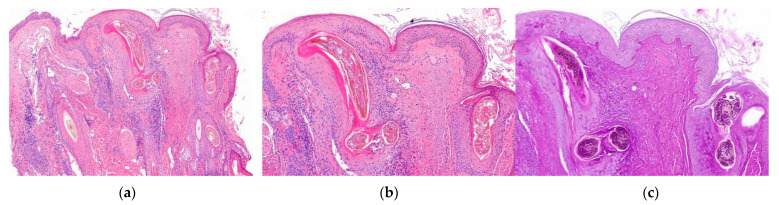
Tinea capitis endothrix type. Vertical sections. (**a**,**b**) Intra and perifollicular inflammation with destruction of the epithelium (HE ×20; HE ×100). (**c**) Visualization of fungi within the hair shaft (PAS ×100).

1. Clinical Features:
  - Common features include scaling, erythema (redness), and hair loss in the affected areas of the scalp.
  - Hair may appear brittle and broken, and there may be evidence of inflammation and crusting.

2. Histological Features (Figure 18):
  - Endothrix—fungi are found inside the hair shaft.
  - Ectothrix—fungi are seen around the hair shaft.
  - Polymorphous inflammatory infiltrate.
  - Damage of the follicular epithelium.
  - Positive fungal stains (PAS, Grocott).

### 5.3. Multifactorial Alopecias

Multifactorial alopecias refer to a group of conditions where the underlying causes of hair loss are a combination of multiple factors. These types of alopecias account for more than 10% of all cases of hair loss and involve a complex interplay of various factors that contribute to the overall condition [85].

## 6. Algorithms

Different algorithms have been developed to aid in the interpretation of histological findings in the context of various clinical presentations. These algorithms provide a systematic approach to diagnosing and classifying different types of alopecia based on histopathological features. We refer our readers to the relevant literature [22,50,86,87].

## 7. Conclusions

Despite the lack of interest in alopecia biopsies by some pathologists, this area of dermatopathology is not more complex than any other. The objective of this article was to briefly review the fundamental notions about the histopathology of the hair follicle and hair cycle, as well as sample management, and provide key diagnostic clinical and histopathological guidelines that will allow pathologists to confidently approach the study of alopecia biopsies.

## Data Availability

Not applicable.
